# Isolation and characterization of *Saccharomyces cerevisiae* mutants with ornithine accumulation for value-added craft beer brewing

**DOI:** 10.1093/jimb/kuag013

**Published:** 2026-05-20

**Authors:** Akira Nishimura, Shota Isogai, Koya Yamada, Ryoya Tanahashi, Hiroshi Takagi

**Affiliations:** Department of Food and Agricultural Sciences, Faculty of Agriculture, Iwate University, 3-18-8 Ueda, Morioka, Iwate 020-8550, Japan; Strategic Initiative for Research and Innovation, Nara Institute of Science and Technology, 8916-5 Takayama, Ikoma, Nara 630-0192, Japan; Graduate School of Science and Technology, Nara Institute of Science and Technology, 8916-5 Takayama, Ikoma, Nara 630-0192, Japan; Department of Food Science and Technology, University of California Davis, One Shields Ave, Davis, CA 95616, USA; Strategic Initiative for Research and Innovation, Nara Institute of Science and Technology, 8916-5 Takayama, Ikoma, Nara 630-0192, Japan

**Keywords:** *ARG6*, craft beer, ornithine, *Saccharomyces cerevisiae*

## Abstract

Ornithine is a nonproteinogenic amino acid with various health benefits, making it a promising target for functional food development. In this study, we developed the yeast *Saccharomyces cerevisiae* strains with elevated intracellular ornithine and identified the genetic mutation responsible for this trait. An ornithine-rich mutant was successfully isolated by chemical mutagenesis and selection for resistance to canavanine, a toxic arginine analog. Whole-genome sequencing revealed a heterozygous point mutation in the *ARG6* gene encoding *N*-acetylglutamate kinase, a key enzyme in ornithine biosynthesis. This mutation caused a Gly351Asp substitution located in a conserved linker region between functional domains. Introduction of this substitution, along with introducing alternative amino acids at the same site, consistently increased ornithine levels in various strain backgrounds. Structural modeling suggested that this substitution could affect the enzyme conformation or inter–domain interactions. These results establish a practical nongenetically modified breeding strategy for enhancing ornithine production in yeast, which will facilitate the development of ornithine-enriched fermented beverages such as craft beer.

**One-sentence summary** The *ARG6* mutant increases intracellular ornithine and enables value-added craft beer brewing.

## Graphical Abstract

**Graphical Abstract:**
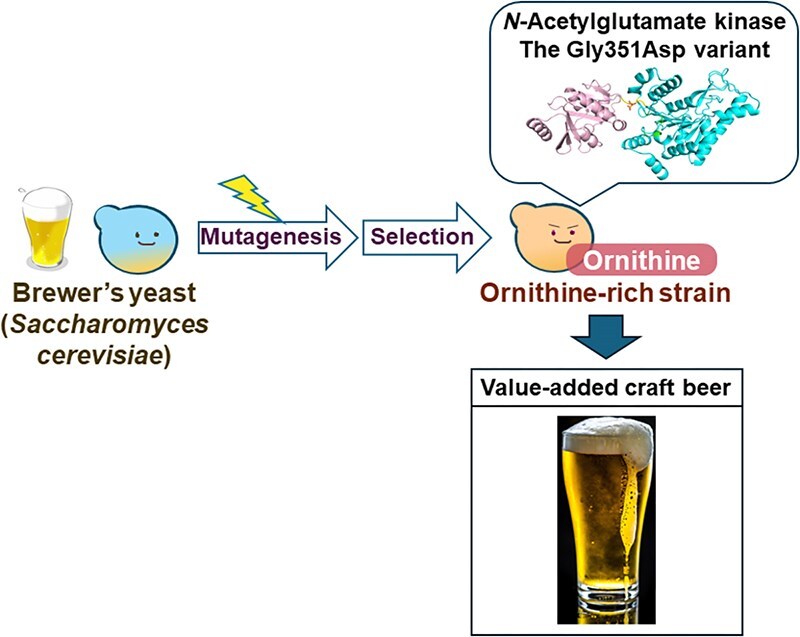

The diversification of craft beer has increasingly extended beyond flavor and regional identity to include added nutritional and functional value. In response to consumer interest in health-conscious products, recent efforts have explored the potential of brewing yeast not only as a fermentation agent but also as a biological source of value-added metabolites (Gobbi et al., 2024; Paiva et al., 2021). Among these strategies, modulation of yeast metabolism to enrich specific bioactive compounds offers a promising route for developing functional fermented beverages.

In fermented foods and beverages, amino acids represent a major class of metabolites that contribute not only to taste and aroma but also to physiological effects in humans. Beyond their role as protein constituents, amino acids participate in diverse regulatory and signaling processes (Wu, 2009). Ornithine, a nonproteinogenic amino acid, has attracted particular attention because of its involvement in multiple biological functions. It plays a central role in ammonia detoxification, serves as a precursor for polyamine biosynthesis, and has been associated with benefits related to fatigue reduction, immune modulation, and tissue maintenance (Bae et al., 2018, Cruzat et al., 2014; Gordon, 2003; Kokubo et al., 2013; Ling et al., 2023; Miura et al., 2023; Yang et al., 2025). Owing to these properties and its established safety, ornithine has been widely incorporated into dietary supplements and functional foods. Extending this concept to fermented beverages, however, requires effective strategies to enhance ornithine production directly within yeast cells.

In the yeast *Saccharomyces cerevisiae*, ornithine is produced in mitochondria as an intermediate in the arginine biosynthetic pathway originating from glutamate (Nishimura et al., 2010; Ohashi et al., 2020; Qin et al., 2015) (Figure S1). Glutamate is first converted into *N*-acetylglutamate (NAG) by *N*-acetylglutamate synthase (Arg2). Subsequently, NAG is phosphorylated by *N*-acetylglutamate kinase (NAGK) to yield *N*-acetylglutamyl-5-phosphate. The *ARG5,6* gene encodes a mitochondrial polyprotein that is post-translationally cleaved to produce two distinct enzymes: NAGK (Arg6) and *N*-acetylglutamyl-5-phosphate reductase (Arg5) (Gessert et al., 1994). Arg2 and Arg6 form a functional complex that catalyzes the initial steps of ornithine biosynthesis. The resulting intermediate is reduced by Arg5, converted from *N*-acetylglutamate semialdehyde to *N*-acetylornithine by acetylornithine aminotransferase (Arg8), deacetylated by Arg7 to form ornithine, and then converted into citrulline by ornithine transcarbamylase (Arg3). Citrulline is further converted into argininosuccinate via argininosuccinate synthase (Arg1) and finally into arginine via argininosuccinate lyase (Arg4). Carbamoyl phosphate, a key precursor in this process, is synthesized by the carbamoyl phosphate synthetase complex composed of Cpa1 and Cpa2 (Delbecq et al., 1994). Excess arginine is converted into ornithine and urea in the cytoplasm by arginase (Car1), linking nitrogen recycling via biosynthesis and catabolism (Kovari et al., 1993).

Regulation of ornithine and arginine metabolism in yeast is achieved through multiple, interconnected mechanisms. Enzymatic activity of the Arg2–Arg6 complex is subject to feedback inhibition by arginine, effectively limiting metabolic flux under arginine-replete conditions (Pauwels et al., 2003). In addition, translational regulation via an upstream open reading frame in *CPA1* and transcriptional control mediated by the ArgR regulatory complex, composed of Arg80, Arg81, and Arg82, further fine-tune pathway activity in response to intracellular arginine levels (Delbecq et al., 1994; Jamai et al., 2002). These regulatory layers are integrated with global nitrogen control systems such as nitrogen catabolite repression, which responds to environmental nitrogen availability (Dubois & Messenguy, 1997). As a consequence, targeted elevation of ornithine levels in *S. cerevisiae* is difficult to achieve, particularly when nongenetically modified (non-GM) approaches are required for food and beverage applications.

In the present study, we isolated a yeast mutant exhibiting markedly increased intracellular ornithine accumulation through chemical mutagenesis and phenotypic selection. Genome analysis identified a causative point mutation in the *ARG6* gene. Importantly, the resulting strain retained normal fermentation performance, enabling the production of ornithine-enriched beer. These findings uncover a previously unrecognized regulatory element in yeast ornithine biosynthesis and demonstrate the feasibility of non-GM breeding strategies for the development of value-added craft beers.

## Materials and methods

### Culture media

The growth media used in this study were a synthetic minimal medium with allantoin (SD-N + allantoin) (2% glucose, 0.17% yeast nitrogen base without any nitrogen sources [Difco Laboratories, Detroit, MI, USA], and 0.5% allantoin as a sole nitrogen source), a yeast extract, peptone, and dextrose medium (YPD) (2% glucose, 1% yeast extract [Difco Laboratories], and 2% peptone [Difco Laboratories]), and a wort medium (11.4% dried malt extract [Briess Malt & Ingredients, Chilton, WI, USA]). All media were adjusted to pH 6.5. When necessary, 300 μg/mL G418 (Nacalai Tesque, Kyoto, Japan) and/or 2% agar (Nacalai Tesque) were added.

### Strains

Table S1 summarizes the yeast strains used in this study. Strain ADH837 of *S. cerevisiae* was originally isolated from a natural environment through a collaborative project between Nara Institute of Science and Technology and 10 Fields Factory Co., Ltd. (Kyoto, Japan), and is currently used commercially by 10 Fields Factory Co., Ltd. The Nottingham Ale, Diamond Lager, and Lalvin L2056 strains were purchased from Lallemand (Montréal, Canada).

### Plasmids

Table S2 lists the oligonucleotide primers used in this study. To construct yeast expression plasmids for *ARG5,6*, genomic DNA isolated from either strain ADH837 or ADHorn49 was used as a PCR template along with gene-specific primers to amplify the *ARG5,6* coding region, including approximately 1 kb of upstream and downstream flanking sequences. The resulting PCR products were ligated into the *Hin*dIII- and *Bam*HI-linearized pYC130 plasmid using the In-Fusion cloning system (Takara Bio, Shiga, Japan), yielding pYC130-*ARG5,6*^WT and pYC130-*ARG5,6*^G351D constructs. To generate Arg6 variants with substitutions at Gly351 (G351A, G351C, G351E, G351F, G351H, G351I, G351K, G351L, G351M, G351N, G351P, G351Q, G351R, G351S, G351T, G351V, G351W, and G351Y), site-directed mutagenesis was performed using the QuikChange method (Agilent Technologies, Santa Clara, CA, USA) with pYC130-*ARG5,6*^WT as the template and mutation-specific primers (Table S2). After DpnI digestion to eliminate the parental plasmid, the reaction mixtures were transformed into *Escherichia coli*. Plasmids were extracted from the transformants to obtain the pYC130-*ARG5,6*^G351X series (X = any amino acid except glycine).

### Chemical mutagenesis

Strain ADH837 was cultured in YPD medium at 30 °C with shaking for 24 h. The cells were then harvested and resuspended in 100 mM phosphate buffer to an optical density at 600 nm (OD₆₀₀) of 3.0. Ethyl methanesulfonate (EMS) was added to the suspension at a final concentration of 5.5–6.5%, followed by incubation at 30 °C for 1 h without shaking. After treatment, cells were collected by filtration and washed with 100 mM phosphate buffer. The washed cells were plated onto YPD agar, and the number of colonies was counted. Cell viability was calculated as the percentage of colonies formed relative to the no-EMS control (set as 100%).

### Isolation of canavanine-resistant mutants

Following EMS mutagenesis, ~4 × 10⁸ cells of ADH837 were spread onto SD-N + allantoin medium supplemented with 150 μg/mL canavanine and incubated at 30 °C for 3–4 days. Colonies that emerged under these selective conditions were individually picked and re-streaked onto fresh SD-N + allantoin medium containing canavanine to verify their resistance phenotype. Strains that consistently demonstrated robust growth were classified as canavanine-resistant mutants and designated as ADHorn strains.

### Amino acid determination

Intracellular amino acids were extracted by resuspending yeast cells (equivalent to OD₆₀₀ = 40) in 1 mL of distilled water, followed by heat treatment at 100 °C for 15 min. After centrifugation, the supernatant was mixed with an equal volume of lithium citrate buffer (pH 2.0). For extracellular amino acids, culture supernatants were obtained by centrifugation and mixed with two- to five-fold volumes of lithium citrate buffer (pH 2.0). All samples were analyzed using a JLC-500/V2 amino acid analyzer (JEOL Ltd., Japan), calibrated with a standard amino acid mixture (Wako Pure Chemical Industries, Japan). Intracellular amino acid levels were normalized using OD₆₀₀ values.

### Whole-genome sequencing

Strains ADH837 and ADHorn49 were grown in YPD medium and then harvested. Genomic DNA was extracted using the Dr. GenTLE (from Yeast) high recovery kit (Takara Bio). Libraries for sequencing were prepared using the NEBNext Ultra DNA Library Prep Kit (New England Biolabs, Ipswich, MA, USA), and paired-end short reads of 150 bp were generated using the Illumina NovaSeq 6000 platform (Illumina, San Diego, CA, USA). Sequencing was performed by a commercial provider (Rhelixa, Tokyo, Japan).

### Structural modeling of Arg6 variants at residue 351

The predicted three-dimensional structures of Arg6 variants (Gly351Asp, Gly351Arg, and Gly351Trp) were generated by homology modeling using SWISS-MODEL (https://swissmodel.expasy.org), with the crystal structure of wild-type Arg6 (PDB ID: 3ZZI) as the template. Structural models of both wild-type and mutant Arg6 proteins were visualized using PyMOL (https://www.pymol.org).

### Structural prediction of the Arg2–Arg6 complex

The predicted structure of the Arg2–Arg6 complex was generated using AlphaFold version 3.0 (https://www.alphafoldserver.com). The predicted complex model was also visualized and analyzed using PyMOL.

### Fermentation test

Yeast strains were inoculated into wort medium at an OD₆₀₀ of 0.1. Fermentation was carried out at 22 °C under static conditions for 4 days. The progression of fermentation was monitored by measuring the cumulative volume of CO₂ evolved using a Fermograph II apparatus (Atto, Tokyo, Japan).

### Statistical analysis

Statistical significance was evaluated using an unpaired Student’s *t*-test for pairwise comparisons, or one-way/two-way analysis of variance (ANOVA) followed by Tukey’s post hoc test for multiple group comparisons. All statistical analyses were performed using Prism 7 (GraphPad Software, Boston, MA). A *p*-value < .05 was considered statistically significant.

## Results and discussion

### Development of an ornithine-overproducing yeast strain by canavanine-based selection

To enhance the nutritional and functional value of craft beer, we aimed to develop *S. cerevisiae* strains capable of overproducing ornithine, a nonproteinogenic amino acid with multiple physiological benefits (Wu, 2009). Because ornithine is a key intermediate in arginine biosynthesis, we hypothesized that arginine-overproducing mutants could serve as a source of ornithine-overproducing strains. Based on this rationale, we employed a selection strategy using canavanine, a toxic arginine analog, to enrich for mutants with altered arginine metabolism.

Optimization of mutagenesis conditions revealed that treatment with 6% EMS yielded a survival rate of 20%–30%, which is within the empirically acceptable range for inducing random mutations (Figure S2). Following EMS-induced mutagenesis of the brewer’s yeast strain ADH837, we screened for canavanine-resistant mutants and isolated 534 clones capable of growing on selective medium (Figure S3). Among these, 140 strains exhibited robust and reproducible growth and were designated as the ADHorn series.

### Isolation of an ornithine-rich yeast mutant strain

**Figure 1:**
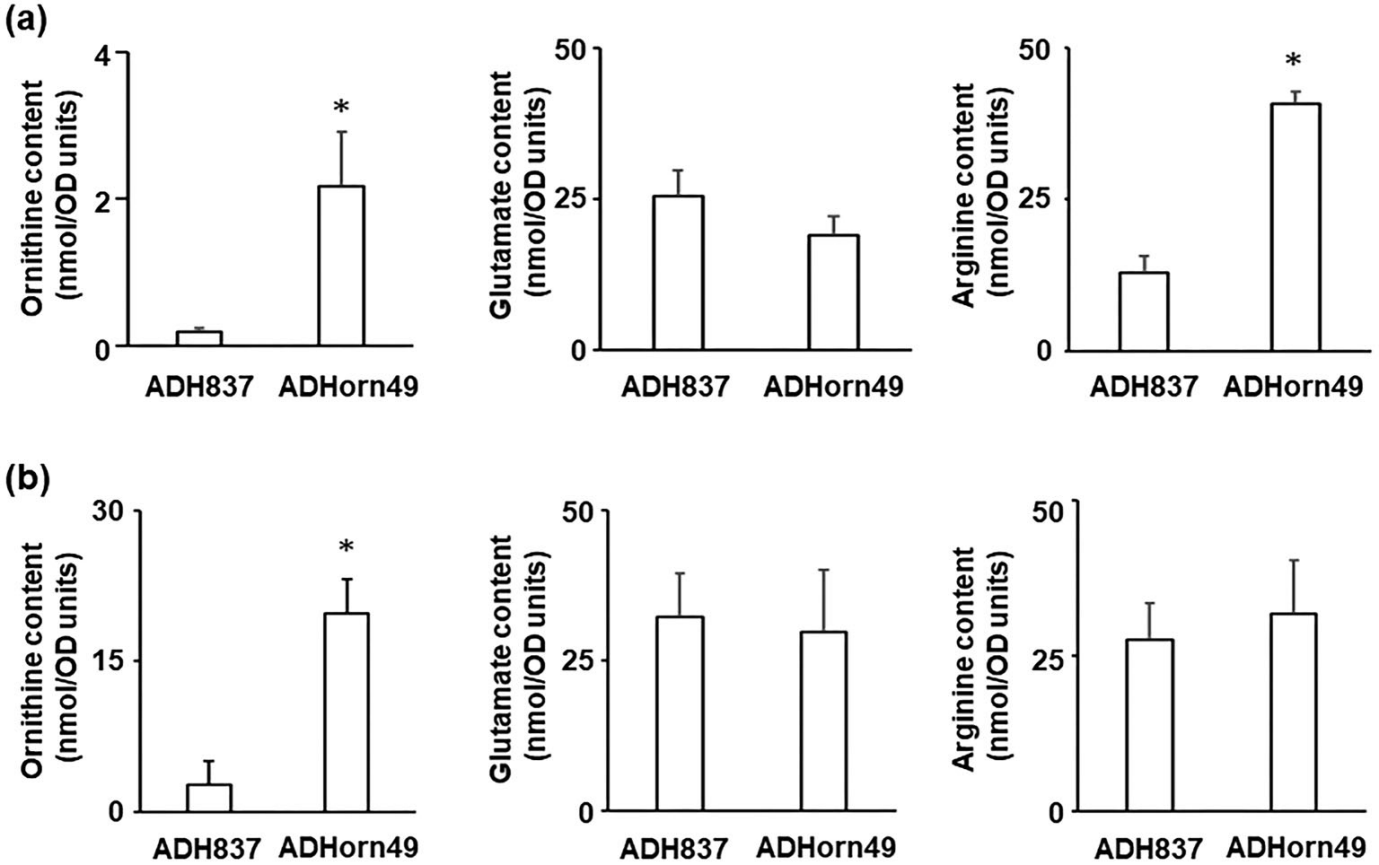
Amino acid accumulation in the ornithine-overproducing mutant ADHorn49. Intracellular levels of ornithine, glutamate, and arginine were determined for the parental strain ADH837 and the mutant strain ADHorn49 after cultivation in SD-N + allantoin medium (a) or YPD medium (b). Values were normalized to optical density (nmol/OD units). Data are presented as mean ± SD from biological replicates (*n* = 3). Statistical significance was assessed using Student’s *t*-test (*p < .05 vs. ADH837).

Intracellular amino acid profiling identified a mutant strain, ADHorn49, with intracellular ornithine levels more than ninefold higher than those of the parental strain (Figure S4). This phenotype was confirmed under minimal (SD) and rich (YPD) medium conditions, showing 8.5-fold and 11.8-fold increases, respectively (Figure 1a and b). Arginine levels remained unchanged in YPD medium but increased 3.1-fold in SD medium. Glutamate levels were unaffected (Figure 1a and b), suggesting that the difference in ornithine accumulation was not attributable to uneven extraction efficiency.

Given the stringent transcriptional and allosteric regulation of the arginine biosynthetic pathway in *S. cerevisiae* (Dubois & Messenguy, 1991; Pauwels et al., 2003), the magnitude of ornithine accumulation observed in ADHorn49 is notable and suggests disruption of a key regulatory mechanism.

### A point mutation in the *ARG6* gene responsible for enhanced ornithine accumulation

Whole-genome sequencing analysis of strain ADHorn49 revealed 327 missense mutations. Among these, a heterozygous G→A transition at position 1 052 in the *ARG5,6* gene was identified as a potential driver mutation. The *ARG5,6* gene encodes a polyprotein that is processed into *N*-acetylglutamate kinase (Arg6) and *N*-acetylglutamyl-5-phosphate reductase (Arg5), both essential for ornithine biosynthesis (Pauwels et al., 2003) (Figure S5).

**Figure 2:**
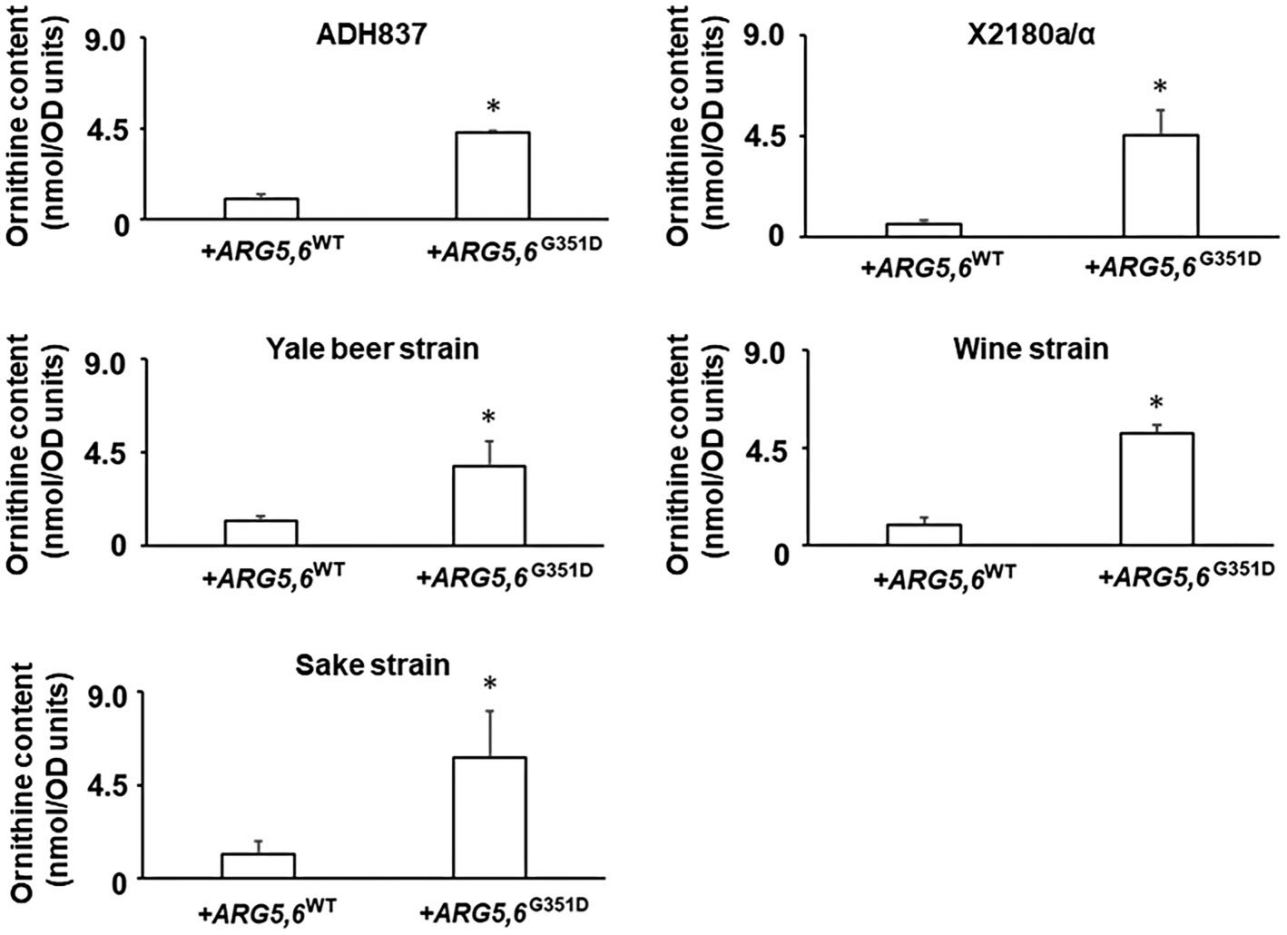
Effect of the G351D substitution of Arg6 on ornithine accumulation across various yeast genetic backgrounds. Multiple yeast strains with different genetic backgrounds, ADH837, X2180a/α (laboratory strain), Yale beer, wine, and sake strains, were transformed with plasmids encoding either the WT or G351D-substituted *ARG5,6* gene and cultured in YPD medium supplemented with G418. Intracellular ornithine concentrations were quantified and normalized to optical density (nmol/OD units). Data are presented as mean ± SD (*n* = 3). Statistical significance was assessed using Student’s *t*-test (*p < .05 vs. WT).

The mutation results in a Gly351Asp substitution in Arg6. This glycine residue is highly conserved among fungal species (Figure S6) and is located within the linker region between the catalytic AAK domain and the fungal-specific domain (Figure S7a and b). Introduction of the mutated *ARG6* allele into multiple industrial yeast strains consistently led to elevated intracellular ornithine levels, confirming that the Gly351Asp substitution is sufficient to confer the high-ornithine phenotype (Figure 2). In industrial diploid background strains, such partial deregulation may be advantageous if it improves ornithine production without compromising fermentation performance.

**Figure 3:**
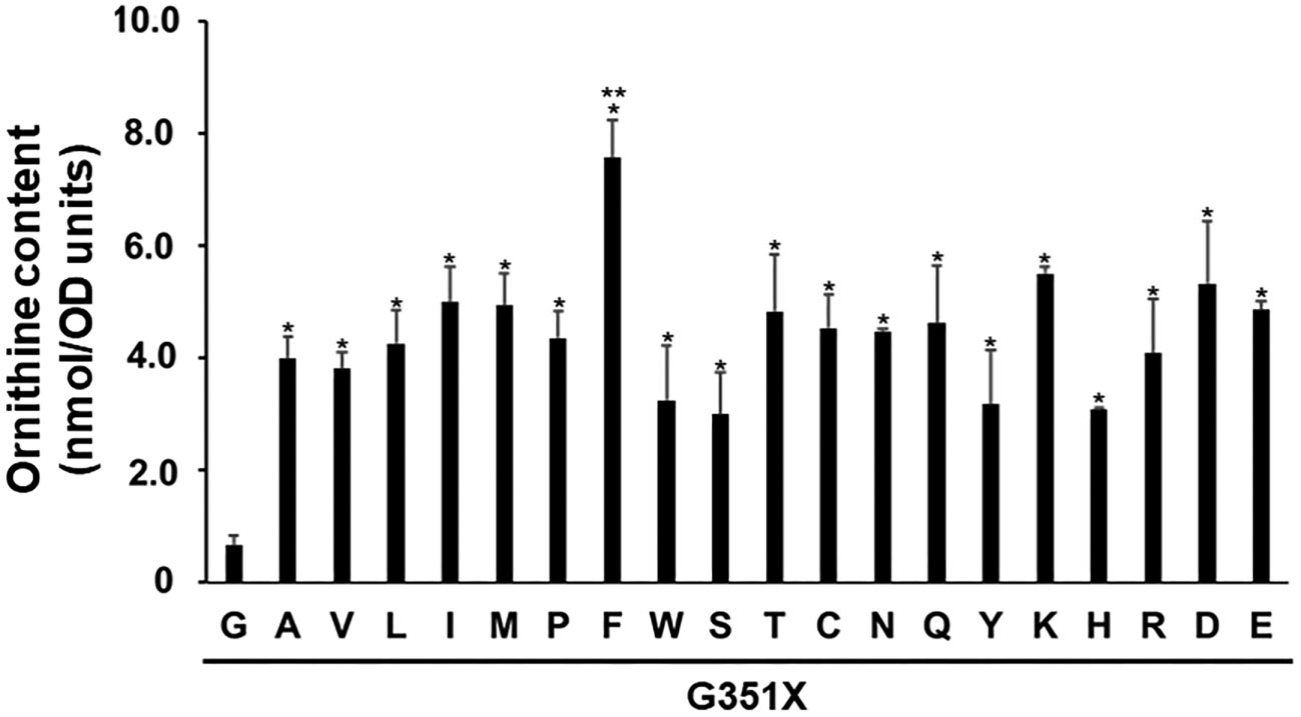
Ornithine production by yeast strains expressing the *ARG5,6* mutant genes. Yeast cells expressing wild-type or mutant *ARG5,6* alleles were cultured in YPD medium supplemented with G418 for 24 h. Intracellular ornithine concentrations were quantified and normalized to optical density (nmol/OD units). The underlying quantitative data for the Gly351 substitution series are provided in Table S3. Data are presented as mean ± SD (n = 3). Statistical significance was determined by one-way ANOVA followed by Tukey’s post hoc test (*p < .05 vs. G; **p < .05 vs. D). Quantitative data corresponding to the Gly351 substitution series are shown in Table S3.

To further assess the functional role of Gly351, this residue was systematically replaced with all other 19 amino acids. Remarkably, all resulting variants, when co-expressed with *ARG5*, led to increased intracellular ornithine accumulation (Figure 3 and Table S3). Among these variants, only Gly351Phe showed a significantly higher intracellular ornithine level than Gly351Asp. These results indicate that Gly351 functions as a negative regulatory site in Arg6, likely involved in arginine-mediated feedback inhibition.

### Structural implications of the Gly351 substitution in Arg6 regulation

**Figure 4:**
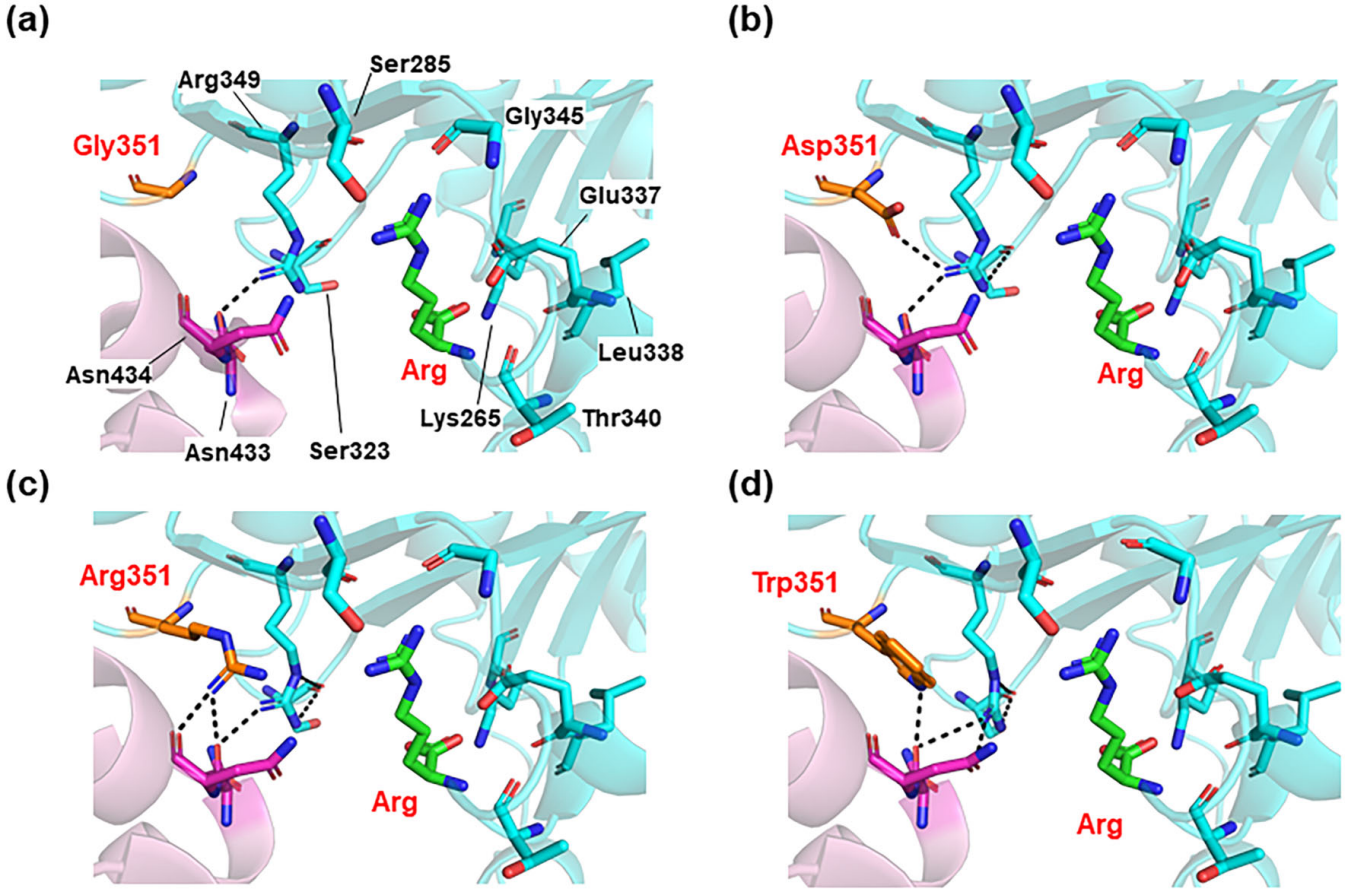
Structural modeling of Arg6 variants carrying substitutions at Gly351. Predicted local conformations surrounding residue 351 are shown for wild-type (a) and the G351D (b), G351R (c), and G351W (d) variants of Arg6. In panel (a), Gly351 and the allosteric inhibitor arginine are shown as orange and green sticks, respectively. Key residues involved in arginine recognition (Lys265, Ser285, Glu337, Leu338, Thr340, and Gly345) are also shown as sticks, and putative hydrogen bonds are indicated by black dashed lines. In panels (b–d), substituted residues at position 351 and neighboring residues potentially involved in intramolecular interactions are displayed in orange and green, respectively. Residues potentially forming interactions with residue 351 (e.g. Arg349, Asn433, and Asn434), along with the arginine-binding residues, are shown as sticks. Putative hydrogen bonds are indicated by black dashed lines.

Gly351 is spatially distant from both the substrate (NAG) and arginine-binding sites in the crystal structure of Arg6 (PDB ID: 3ZZI) (de Cima et al., 2012) (Figure S7b), suggesting an indirect regulatory mechanism. For structural analysis, we modeled Gly351Asp together with Gly351Arg and Gly351Trp. Gly351Arg and Gly351Trp were selected as representative substitutions with distinct side-chain properties to examine whether different types of replacement at this position might produce similar structural effects. Structural modeling revealed that mutations at this position introduced new hydrogen-bonding networks involving Arg349 in the AAK domain and Asn433 and Asn434 in the fungal-specific domain (Figure 4a–d). These interactions are predicted to enhance interdomain contacts, potentially stabilizing a conformation that is less sensitive to arginine-mediated feedback inhibition.

**Figure 5:**
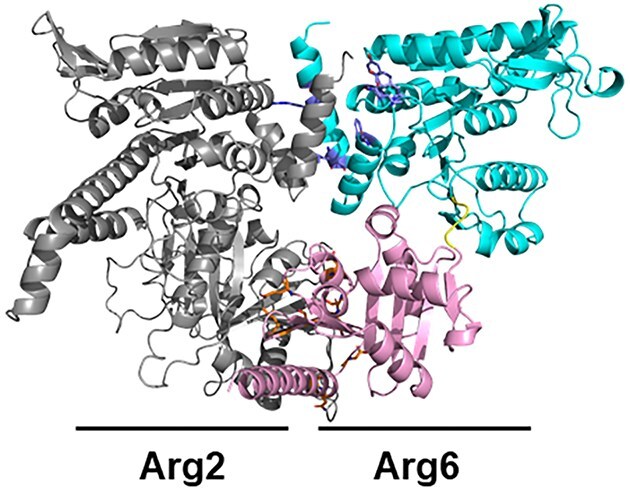
Predicted structures of the Arg2–Arg6 complex. A structural model of the Arg2–Arg6 complex was generated by AlphaFold version 3.0. Arg2, the catalytic domain of Arg6, and the fungal-specific domain of Arg6 are depicted as distinct cartoon representations in dark gray, cyan, and magenta, respectively.

Previous studies have shown that Arg6 is regulated not only transcriptionally by the ArgR–Mcm1 complex but also allosterically by arginine binding (Dubois & Messenguy, 1991; Pauwels et al., 2003). In addition, Arg6 is known to form a functional complex with Arg2, mediated in part by the fungal-specific domain (de Cima et al., 2012). Modeling of the Arg2-Arg6 complex in this study predicts that Arg6 interacts with Arg2 via both N-terminal residues of the AAK domain and C-terminal residues of the fungal-specific domain (Figure 5). The Gly351Asp substitution may therefore enhance ornithine production by attenuating feedback inhibition and/or stabilizing the Arg2–Arg6 complex, thereby sustaining enzymatic activity in the ornithine biosynthetic pathway. In particular, the consistent increase in ornithine accumulation across the entire series of substitution suggests that this phenotype is not due to the specific properties of the side chain, but rather reflects the loss of glycine at this position. Since Gly351 is located in the linker region distant from the arginine-binding pocket, a substitution at this site may affect local flexibility or interdomain interactions, potentially leading to reduced sensitivity to arginine-mediated feedback inhibition. Additionally, effects on the stability of the Arg2–Arg6 complex may also be involved. Further biochemical and structural analyses will likely be necessary to distinguish among these possibilities.

### Brewing performance and application potential of strain ADHorn49

**Figure 6:**
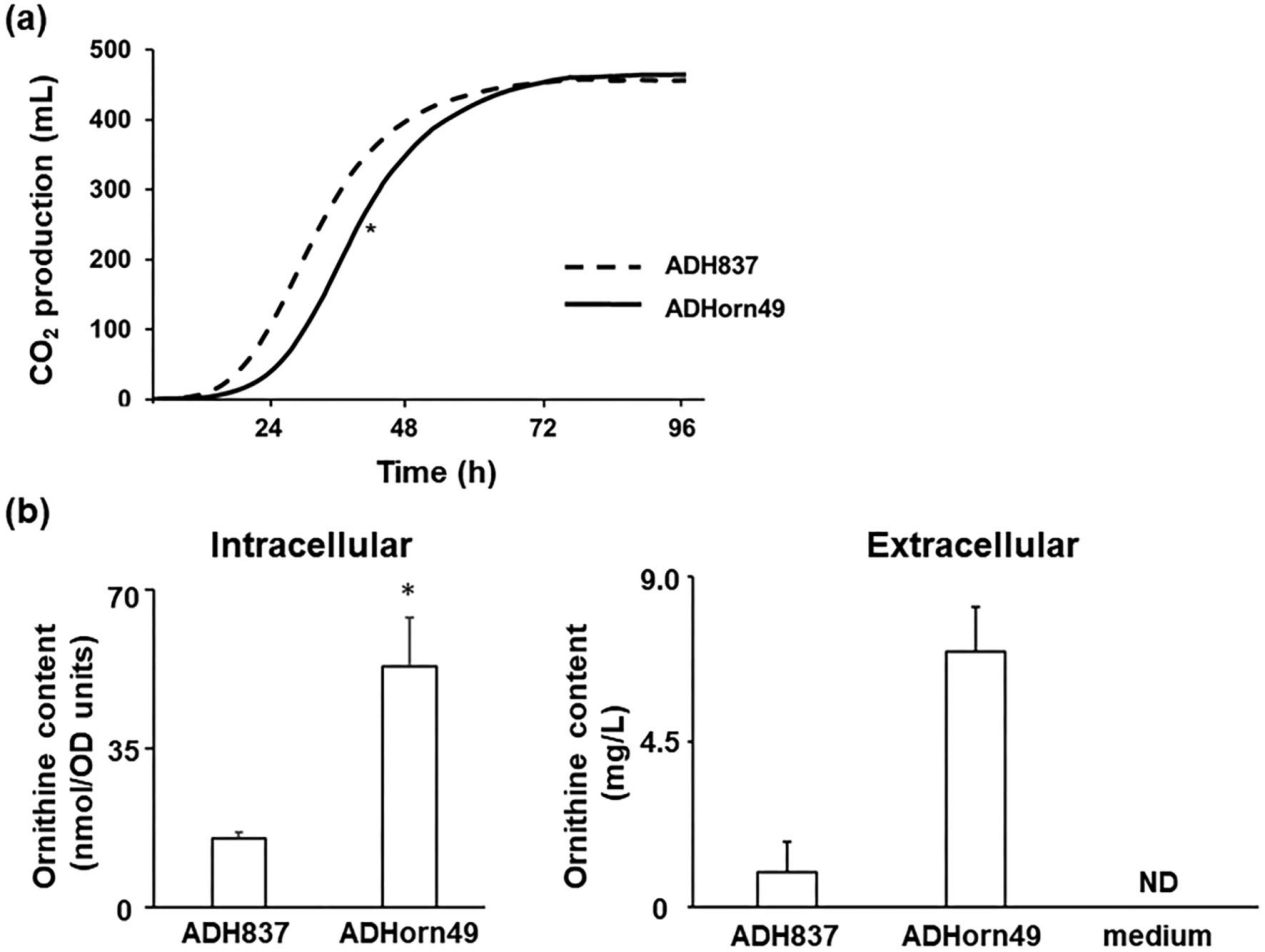
Fermentation performance and ornithine accumulation during static wort fermentation. (a) Carbon dioxide release during fermentation in wort medium under static conditions was monitored over time using a Fermograph. A representative profile from three independent experiments is shown. Statistical significance was determined by two-way ANOVA followed by Tukey’s post hoc test (*p < .05 vs. ADH837). (b) Intracellular (left) and extracellular (right) ornithine levels were measured after 96 h of static fermentation. Data are presented as mean ± SD (*n* = 3). Statistical significance was assessed using Student’s *t*-test (*p < .05 vs. ADH837). ND indicates values below the detection limit.

To evaluate the industrial applicability of strain ADHorn49, its fermentation performance was assessed in wort medium. CO₂ production profiles of strain ADHorn49 and the parental strain ADH837 were indistinguishable, indicating comparable fermentative activity (Figure 6a). The slight difference in CO₂ production observed during the early stages of fermentation was statistically significant, the difference was small and did not affect the overall performance of the fermentation. Notably, strain ADHorn49 exhibited a 3.5-fold increase in intracellular ornithine levels, accompanied by a significant increase in ornithine secretion into the broth after 96 h of wort fermentation, while arginine levels remained unchanged (Figure 6b). The extracellular ornithine concentration in the ADHorn49 fermentation broth reached 52.7 nmol/mL, corresponding to 7.0 mg/L free ornithine. Although this level is lower than those used in supplementation studies, it falls within the range reported for some ornithine-containing foods and supports the potential feasibility of enriching a fermented beverage with ornithine through non-GM yeast breeding (Miura et al., 2023; Yang et al., 2025).

These results demonstrate that enhanced ornithine production does not compromise fermentation performance, highlighting the suitability of strain ADHorn49 for brewing applications. Ornithine is known to exert multiple physiological benefits and has attracted attention as a functional ingredient in foods and beverages (Wu, 2009). Moreover, ornithine serves as a biosynthetic precursor for bioactive compounds such as polyamines and tropane alkaloids (Sato et al., 2001). Ornithine-overproducing strains such as ADHorn49 could serve as useful starting points for future pathway engineering toward ornithine-derived compounds.

## Concluding remarks

This study identifies a previously unrecognized regulatory site in yeast ornithine metabolism and demonstrates that single-point mutations in a conserved residue of Arg6 can dramatically enhance intracellular ornithine accumulation. Importantly, strain ADHorn49 was generated through non-GM mutagenesis and selection, offering a practical and scalable strategy for producing ornithine-enriched craft beer and other functional fermented beverages. In addition, strain ADHorn49 provides a versatile platform for future metabolic engineering efforts aimed at producing ornithine-derived bioactive compounds.

## Supplementary Material

kuag013_Supplemental_File

## Data Availability

Whole-genome sequencing data from this study are available in the DDBJ Sequence Read Archive (https://www.ddbj.nig.ac.jp/index-e.html) under the accession numbers DRR544848 (ADH837) and DRR544850 (ADHorn49).
