# Supplementary material for: Isolation and characterization of Saccharomyces cerevisiae mutants with ornithine accumulation for value-added craft beer brewing
Source: J Ind Microbiol Biotechnol. 2026 May 20;53:kuag013. doi: 10.1093/jimb/kuag013 (PMC13261765; doi:10.1093/jimb/kuag013)
Supplement: kuag013_Supplemental_File [file kuag013_supplemental_file.docx]

**Supplemental materials**

**Isolation and characterization of *Saccharomyces cerevisiae* mutants with ornithine accumulation for value-added craft beer brewing**

**Akira Nishimura^1#^*, Shota Isogai^2#^, Koya Yamada^3^, Ryoya Tanahashi^4^, Hiroshi Takagi^2^****

^1^Department of Food and Agricultural Sciences, Faculty of Agriculture, Iwate University, 3-18-8 Ueda, Morioka, Iwate 020-8550, Japan

^2^Strategic Initiative for Research and Innovation, Nara Institute of Science and Technology, 8916-5 Takayama, Ikoma, Nara 630-0192, Japan

^3^Graduate School of Science and Technology, Nara Institute of Science and Technology, 8916-5 Takayama, Ikoma, Nara 630-0192, Japan

^4^Department of Food Science and Technology, University of California Davis, One Shields Ave, Davis, CA, 95616, USA

*^#^*The first two authors contributed equally to this work.

^*^Corresponding author. Email: nakira@iwate-u.ac.jp, Tel: +81-19-621-6130.

**^**^**Corresponding author. Email: hiro@bs.naist.jp, Tel: +81-743-72-5420.

**This PDF file includes:**

Tables S1–S3

Figs S1–S7

**Table S1. Yeast strains used in this study.**

| Abbreviation | Description |
| --- | --- |
| ADH837 | Private *Saccharomyces cerevisiae* strain |
| ADHorn49 | Ornithine-overproducing mutant derived from strain ADH837 |
| Nottingham Ale | Commercially available ale-brewing yeast strain |
| Diamond lager | Commercially available lager-brewing yeast strain |
| Lalvin L2056 | Commercially available wine yeast strain |
| K7 | Sake-brewing yeast strain |
| X2180a/α | Laboratory strain |

**Table S2. Oligonucleotide primers used in this study.**

| Name | Sequence (5’ 🡪 3’) |
| --- | --- |
| ARG5,6 Fw（EcoRI） | CGACGGTACCGAATTAAGAAACTTATAAAAAGTATCCG |
| ARG5,6 Rv（BamHI） | GCGGCGCCTAGGATCTCTTATCGAATTGGACAGGT |
| ARG5,6（G351A）Fw | ATCTCTTCACTAATTTGTAAGCTCTCCTGATCATAGTACCC |
| ARG5,6（G351A）Rv | GGGTACTATGATCAGGAGAGCTTACAAATTAGTGAAGAGAT |
| ARG5,6（G351C）Fw | TCTCTTCACTAATTTGTAACATCTCCTGATCATAGTACCCG |
| ARG5,6（G351C）Rv | CGGGTACTATGATCAGGAGATGTTACAAATTAGTGAAGAGA |
| ARG5,6（G351E）Fw | GGATCTCTTCACTAATTTGTACTCTCTCCTGATCATAGTACCCG |
| ARG5,6（G351E）Rv | CGGGTACTATGATCAGGAGAGAGTACAAATTAGTGAAGAGATCC |
| ARG5,6（G351F）Fw | GGATCTCTTCACTAATTTGTAAAATCTCCTGATCATAGTACCCGCA |
| ARG5,6（G351F）Rv | TGCGGGTACTATGATCAGGAGATTTTACAAATTAGTGAAGAGATCC |
| ARG5,6（G351H）Fw | GATCTCTTCACTAATTTGTAATGTCTCCTGATCATAGTACCCGC |
| ARG5,6（G351H）Rv | GCGGGTACTATGATCAGGAGACATTACAAATTAGTGAAGAGATC |
| ARG5,6（G351I）Fw | GGATCTCTTCACTAATTTGTAAATTCTCCTGATCATAGTACCCGCA |
| ARG5,6（G351I）Rv | TGCGGGTACTATGATCAGGAGAATTTACAAATTAGTGAAGAGATCC |
| ARG5,6（G351K）Fw | GAGGATCTCTTCACTAATTTGTACTTTCTCCTGATCATAGTACCCGCAC |
| ARG5,6（G351K）Rv | GTGCGGGTACTATGATCAGGAGAAAGTACAAATTAGTGAAGAGATCCTC |
| ARG5,6（G351L）Fw | GAGGATCTCTTCACTAATTTGTATAGTCTCCTGATCATAGTACCCGCAC |
| ARG5,6（G351L）Rv | GTGCGGGTACTATGATCAGGAGACTATACAAATTAGTGAAGAGATCCTC |
| ARG5,6（G351M）Fw | GAGGATCTCTTCACTAATTTGTACATTCTCCTGATCATAGTACCCGCAC |
| ARG5,6（G351M）Rv | GTGCGGGTACTATGATCAGGAGAATGTACAAATTAGTGAAGAGATCCTC |
| ARG5,6（G351N）Fw | GGATCTCTTCACTAATTTGTAATTTCTCCTGATCATAGTACCCGCA |
| ARG5,6（G351N）Rv | TGCGGGTACTATGATCAGGAGAAATTACAAATTAGTGAAGAGATCC |
| ARG5,6（G351P）Fw | GATCTCTTCACTAATTTGTAAGGTCTCCTGATCATAGTACCCGC |
| ARG5,6（G351P）Rv | GCGGGTACTATGATCAGGAGACCTTACAAATTAGTGAAGAGATC |
| ARG5,6（G351Q）Fw | GAGGATCTCTTCACTAATTTGTACTGTCTCCTGATCATAGTACCCGCAC |
| ARG5,6（G351Q）Rv | GTGCGGGTACTATGATCAGGAGACAGTACAAATTAGTGAAGAGATCCTC |
| ARG5,6（G351R）Fw | CTCTTCACTAATTTGTAACGTCTCCTGATCATAGTACCC |
| ARG5,6（G351R）Rv | GGGTACTATGATCAGGAGACGTTACAAATTAGTGAAGAG |
| ARG5,6（G351S）Fw | TCTCTTCACTAATTTGTAACTTCTCCTGATCATAGTACCCG |
| ARG5,6（G351S）Rv | CGGGTACTATGATCAGGAGAAGTTACAAATTAGTGAAGAGA |
| ARG5,6（G351T）Fw | GATCTCTTCACTAATTTGTAAGTTCTCCTGATCATAGTACCCGC |
| ARG5,6（G351T）Rv | GCGGGTACTATGATCAGGAGAACTTACAAATTAGTGAAGAGATC |
| ARG5,6（G351V）Fw | GATCTCTTCACTAATTTGTAAACTCTCCTGATCATAGTACCCG |
| ARG5,6（G351V）Rv | CGGGTACTATGATCAGGAGAGTTTACAAATTAGTGAAGAGATC |
| ARG5,6（G351W）Fw | GGATCTCTTCACTAATTTGTACCATCTCCTGATCATAGTACCCGC |
| ARG5,6（G351W）Rv | GCGGGTACTATGATCAGGAGATGGTACAAATTAGTGAAGAGATCC |
| ARG5,6（G351Y）Fw | GGATCTCTTCACTAATTTGTAATATCTCCTGATCATAGTACCCGCA |
| ARG5,6（G351Y）Rv | TGCGGGTACTATGATCAGGAGATATTACAAATTAGTGAAGAGATCC |

**Table S3. Intracellular ornithine accumulation.**

| **G351X** | **Ornithine content**  **(nmol/OD units, mean±SD)** | **Fold change**  **(relative to G351G)** |
| --- | --- | --- |
| **G** | **0.7±0.2** | **1.0** |
| **A** | **4.0±0.4** | **5.7** |
| **V** | **3.8±0.3** | **5.4** |
| **L** | **4.3±0.6** | **6.1** |
| **I** | **5.0±0.6** | **7.1** |
| **M** | **4.9±0.6** | **7.0** |
| **P** | **4.4±0.5** | **6.3** |
| **F** | **7.6±0.7** | **10.9** |
| **W** | **3.2±1.0** | **4.6** |
| **S** | **3.0±0.8** | **4.3** |
| **T** | **4.8±1.0** | **6.9** |
| **C** | **4.5±0.6** | **6.4** |
| **N** | **4.5±0.1** | **6.4** |
| **Q** | **4.6±1.0** | **6.6** |
| **Y** | **3.2±1.0** | **4.6** |
| **K** | **5.5±0.1** | **7.9** |
| **H** | **3.1±0.0** | **4.4** |
| **R** | **4.1±1.0** | **5.9** |
| **D** | **5.3±1.1** | **7.6** |
| **E** | **4.9±0.2** | **7.0** |

**
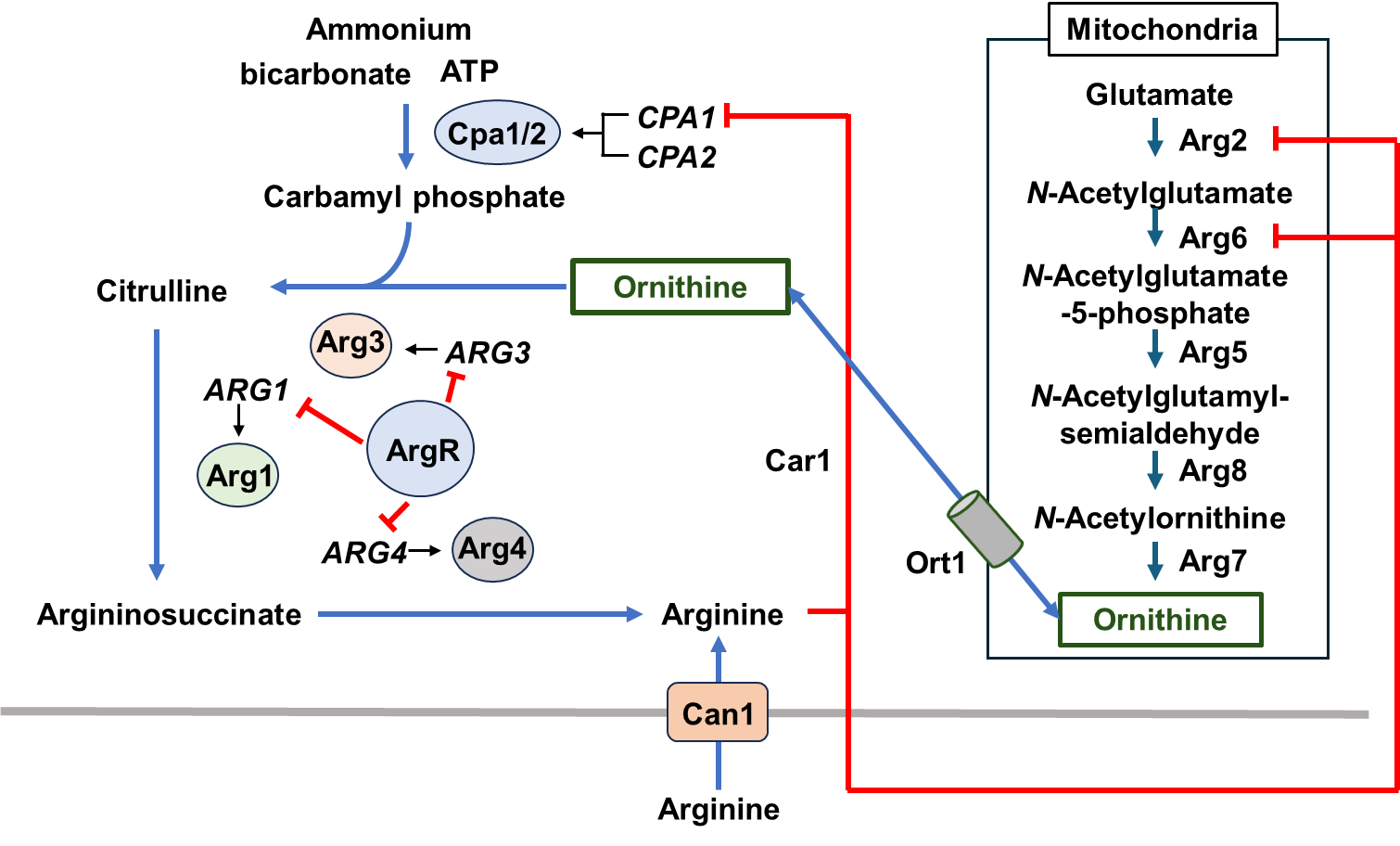
**

**Fig. S1. Ornithine biosynthesis and its regulation in *Saccharomyces cerevisiae*.**

Ornithine is synthesized from glutamate in the mitochondria through a series of enzymatic steps involving *N*-acetylated intermediates. This pathway includes the following enzymes: Arg2 (*N*-acetylglutamate synthase), Arg6 (*N*-acetylglutamate kinase), Arg5 (*N*-acetylglutamate-5-phosphate reductase), Arg8 (Acetylornithine aminotransferase), and Arg7 (*N*-acetylornithinase). The resulting ornithine is transported into the cytoplasm via the mitochondrial ornithine transporter Ort1. In the cytoplasm, ornithine is converted into arginine through the actions of Arg3 (ornithine carbamoyltransferase), Arg1 (argininosuccinate synthase), and Arg4 (argininosuccinate lyase). Extracellular arginine is imported by the transporter Can1. The transcriptional repressor ArgR downregulates the expression of *ARG1*, *ARG3*, and *ARG4* in response to intracellular arginine levels. Arginine also inhibits the enzymatic activity of Arg2 and Arg6. Red lines indicate transcriptional repression or feedback inhibition mediated by arginine.


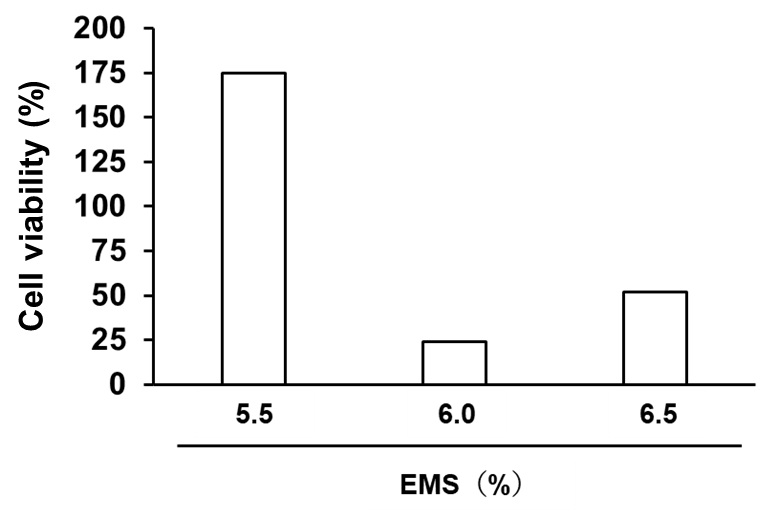


**Fig. S2. Cell viability after EMS treatment.**

Survival rates of yeast cells after exposure to various concentrations of ethyl methanesulfonate (EMS) were determined.

**
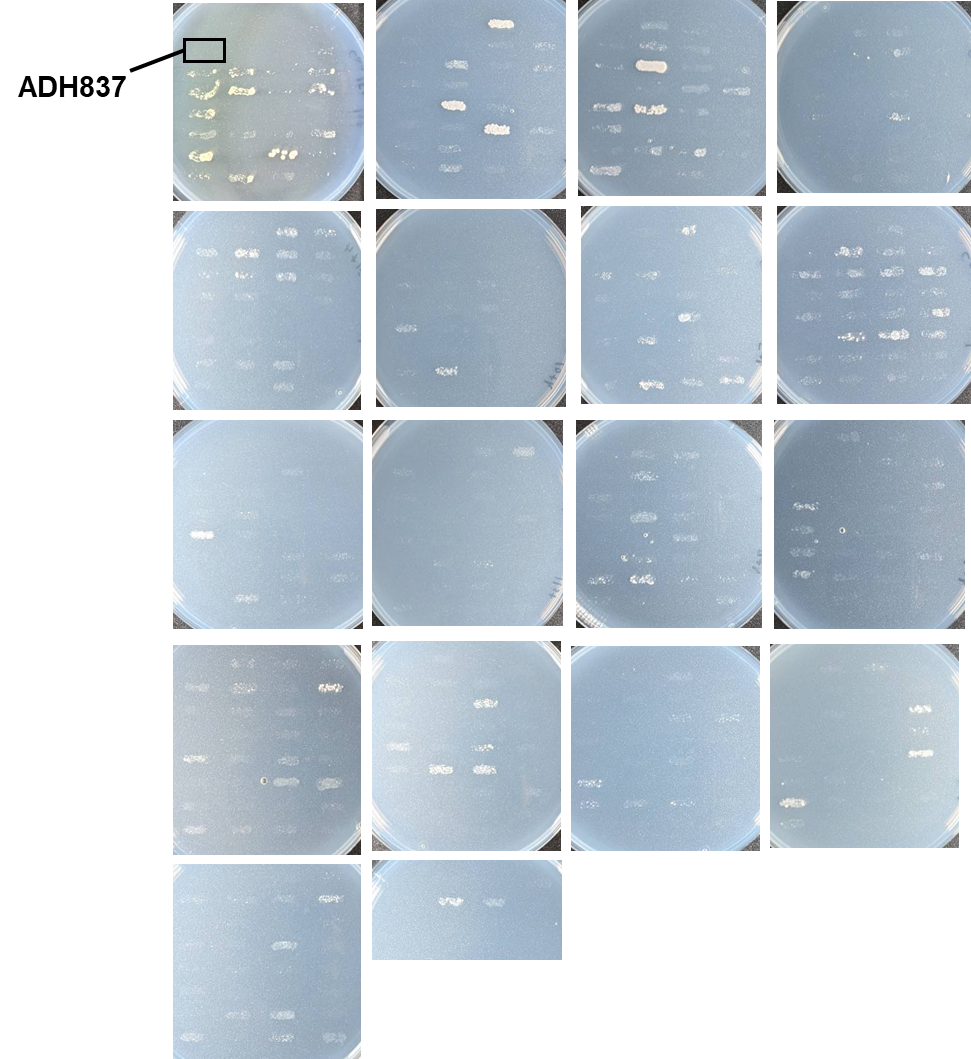
**

**Fig. S3. Screening of canavanine-resistant mutants.**

Out of 534 EMS-treated colonies, 140 strains exhibiting robust and reproducible growth on canavanine-containing medium were selected for further analysis. These mutants, designated as the ADHorn series, were subjected to intracellular amino acid quantification.


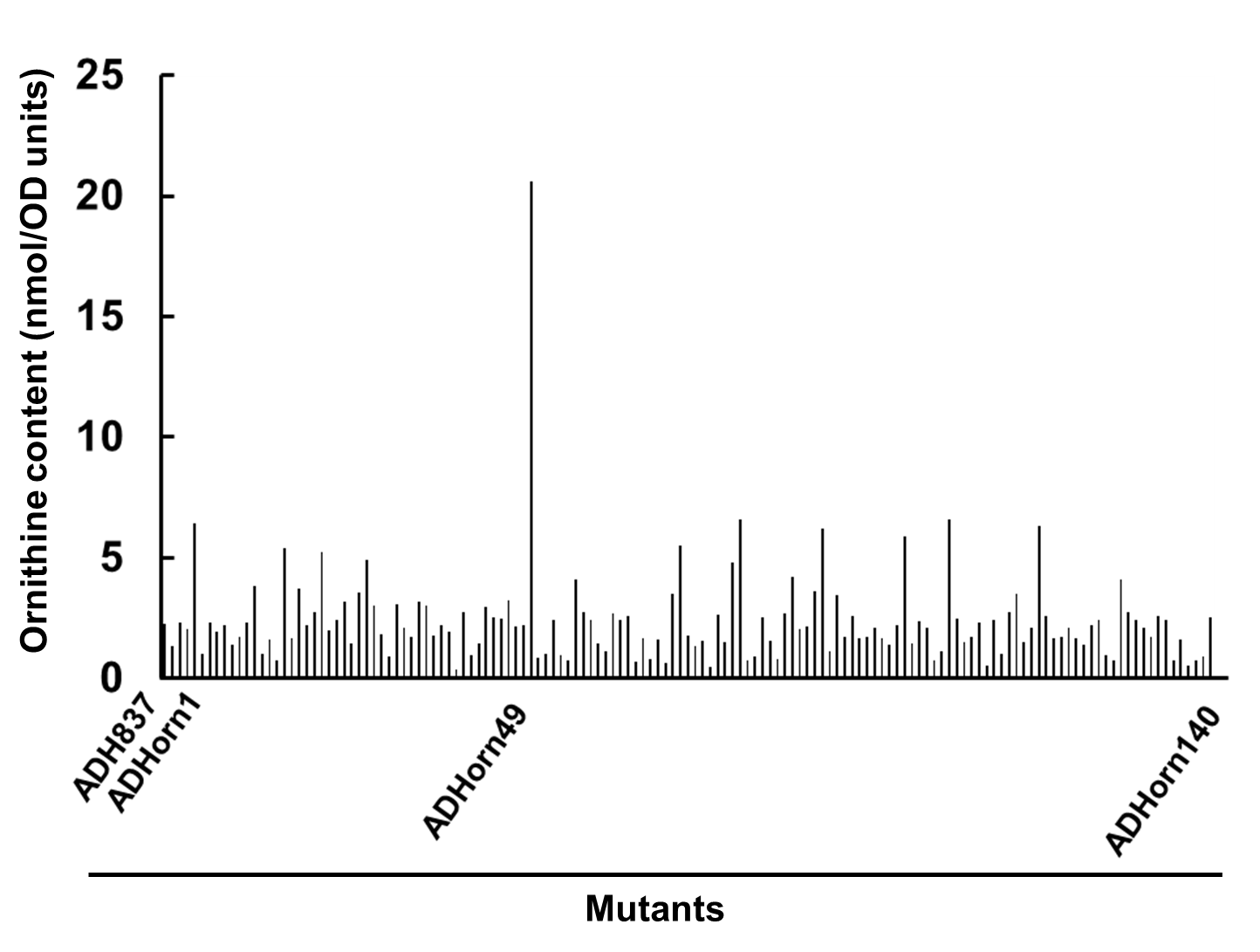


**Fig. S4. Identification of a canavanine-resistant mutant with elevated intracellular ornithine.**

A total of 140 canavanine-resistant mutants were cultivated in YPD medium, and their intracellular ornithine levels were quantified.


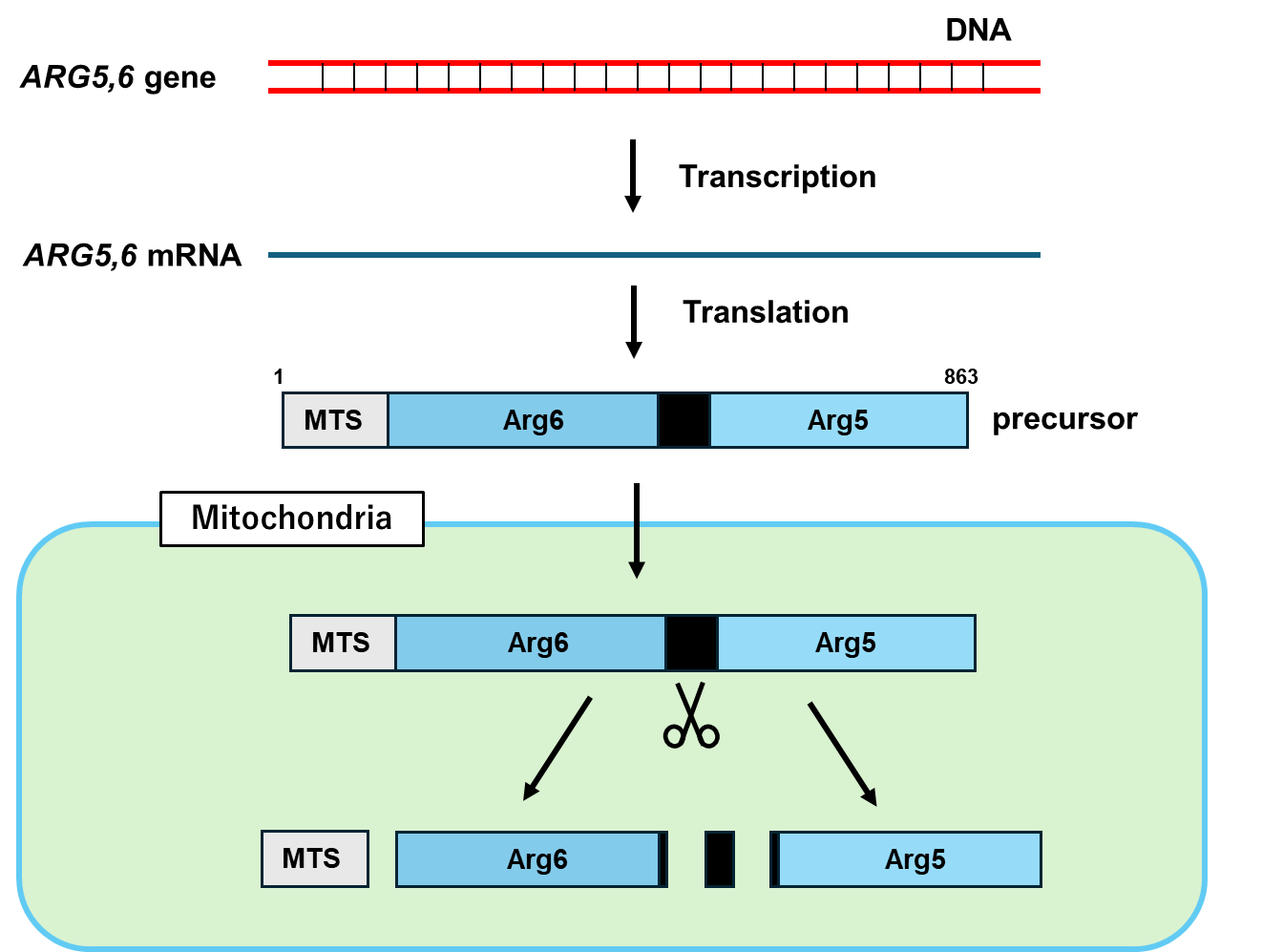


**Fig. S5. Schematic representation of the Arg5,6 precursor and its post-translational processing in *Saccharomyces cerevisiae*.**

The *S. cerevisiae* *ARG5,6* gene encodes a single precursor polypeptide that is imported into mitochondria and subsequently cleaved into two functional enzymes: Arg5 and Arg6. Arg5 exhibits *N*-acetylglutamate kinase (NAGK) activity, while Arg6 functions as *N*-acetylglutamate phosphate reductase (NAGPR), catalyzing consecutive steps in the ornithine biosynthesis pathway. The N-terminal region of the precursor harbors a mitochondrial targeting sequence (MTS), which directs the polypeptide to the mitochondrial matrix prior to its proteolytic cleavage and activation.

**
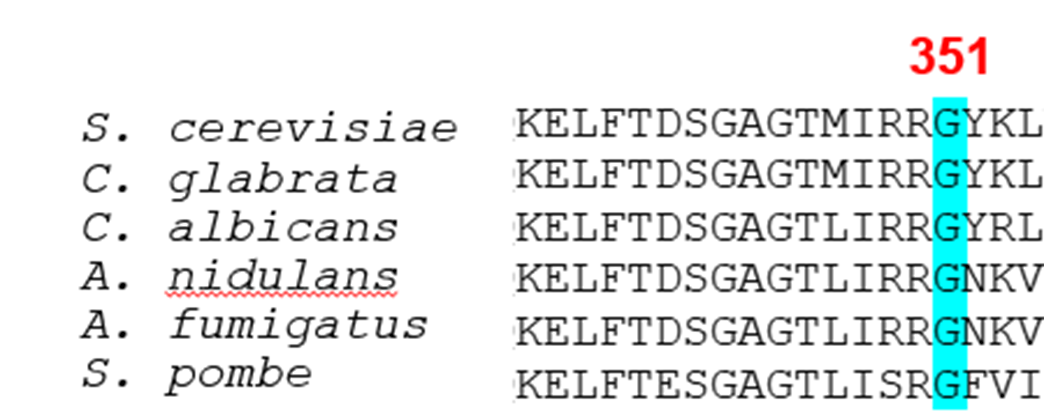
**

**Fig. S6. Multiple sequence alignment of the Gly351-containing region in fungal Arg6 homologs.**

A multiple sequence alignment of Arg6 homologs from representative fungal species, focusing on the region surrounding Gly351 in *S. cerevisiae*. The alignment includes the following species: *Saccharomyces cerevisiae* (*S. cerevisiae*), *Candida glabrata* (*C. glabrate*), *Candida albicans* (*C. albicans*), *Aspergillus nidulans* (*A. nidulans*), *Aspergillus fumigatus* (*A. fumigatus*), and *Schizosaccharomyces pombe* (*S. pombe*).

**
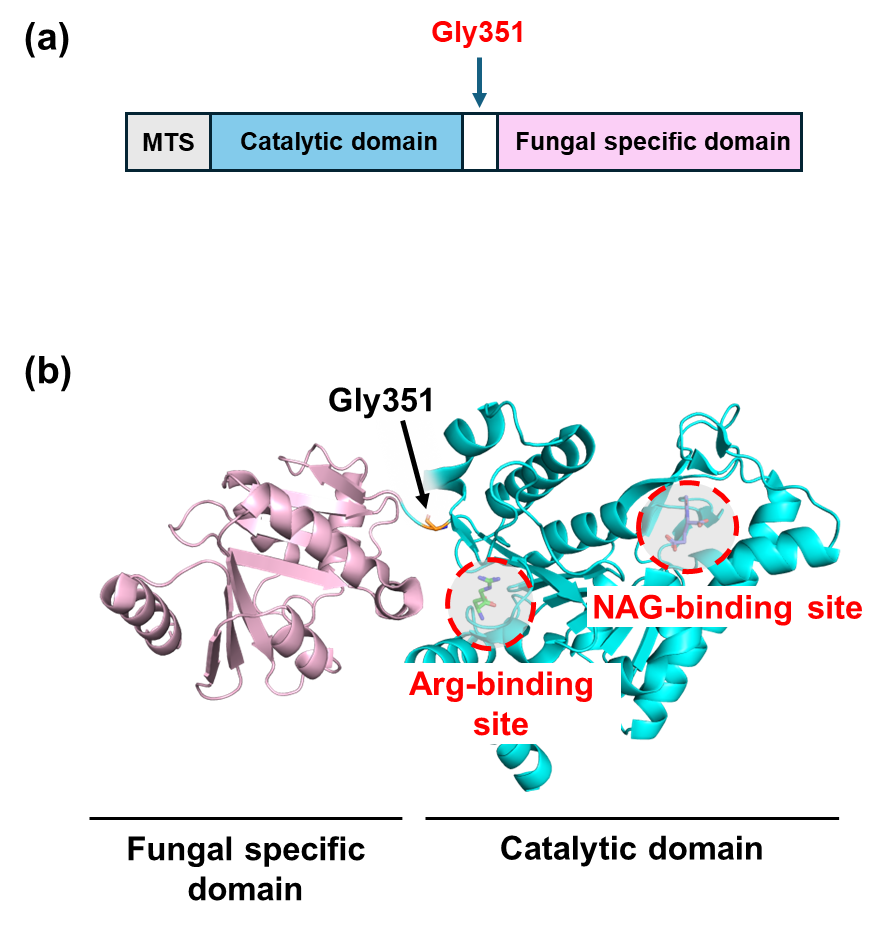
**

**Fig. S7. Domain organization of Arg6 and the position of Gly351.**

(a) Linear domain structure of *S. cerevisiae* Arg6, highlighting the position of Gly351. Arg6 consists of an N-terminal mitochondrial targeting sequence (MTS), a central catalytic domain, and a C-terminal fungal-specific domain. Gly351 is located at the boundary between the catalytic and fungal-specific domains. (b) Overall structure of Arg6. The catalytic domain and fungal-specific domain are shown as cyan and pink cartoons, respectively. Gly351 is indicated in orange stick representation. The binding sites for the substrate *N*-acetylglutamate (NAG) and the allosteric inhibitor arginine, derived from the crystal structure of the catalytic domain (PDB ID: 3ZZH), are highlighted with red circles.
